# Complex Pearson Correlation Coefficient for EEG Connectivity Analysis

**DOI:** 10.3390/s22041477

**Published:** 2022-02-14

**Authors:** Zoran Šverko, Miroslav Vrankić, Saša Vlahinić, Peter Rogelj

**Affiliations:** 1 Faculty of Engineering, Department of Automation and Electronics, University of Rijeka, 51000 Rijeka, Croatia; zsverko@riteh.hr (Z.Š.); miroslav.vrankic@riteh.hr (M.V.); sasa.vlahinic@riteh.hr (S.V.); 2 Faculty of Mathematics, Natural Sciences and Information Technologies, University of Primorska, 6000 Koper, Slovenia

**Keywords:** *EEG*, functional connectivity, phase locking value, weighted phase lag index, complex Pearson correlation coefficients

## Abstract

In the background of all human thinking—acting and reacting are sets of connections between different neurons or groups of neurons. We studied and evaluated these connections using electroencephalography (*EEG*) brain signals. In this paper, we propose the use of the complex Pearson correlation coefficient *(CPCC)*, which provides information on connectivity with and without consideration of the volume conduction effect. Although the Pearson correlation coefficient is a widely accepted measure of the statistical relationships between random variables and the relationships between signals, it is not being used for *EEG* data analysis. Its meaning for *EEG* is not straightforward and rarely well understood. In this work, we compare it to the most commonly used undirected connectivity analysis methods, which are phase locking value (*PLV*) and weighted phase lag index (*wPLI*). First, the relationship between the measures is shown analytically. Then, it is illustrated by a practical comparison using synthetic and real *EEG* data. The relationships between the observed connectivity measures are described in terms of the correlation values between them, which are, for the absolute values of CPCC and PLV, not lower that 0.97, and for the imaginary component of CPCC and wPLI—not lower than 0.92, for all observed frequency bands. Results show that the *CPCC* includes information of both other measures balanced in a single complex-numbered index.

## 1. Introduction

A human brain contains on average about 100 billion ($10^{11}$) neurons connected by about 100 trillion ($10^{14}$) synapses. The neurons are anatomically organized in different spatial regions and functionally interact over different time points [1]. In this work, electroencephalography (*EEG*) was used to record neuron activity. *EEG* is an electrophysiological monitoring method for observing neurophysiological changes related to postsynaptic activity in the neocortex, i.e., a method for recording the electrical activity of the brain [2]. Monitoring brain activity using this method provides high temporal resolution. This property makes *EEG* one of the most suitable monitoring methods for non-invasive detection of neurons’ interactions inside the brain and, consequently, for detection of information transmission within the same brain regions and between different brain regions [3]. Brain connectivity analysis is generally divided into two types: structural and functional. Tracking the direction of fibers between different brain regions or within a brain region is called structural connectivity analysis [4]. The most suitable recording methods for determining structural connectivity are magnetic resonance imaging (*MRI*) [5] and diffusion tensor imaging (*DTI*) [6]. On the other hand, functional connectivity analysis can be defined as an analysis of the amount of information transmitted between brain regions or within a brain region. This type of connectivity analysis is usually divided into two groups: undirected and directed. Undirected connectivity measures evaluate the degree of connectivity, while directed connectivity measures evaluate the degree and direction of connectivity between observed brain regions. In this paper, we focus on undirected connectivity measures. Different cognitive tasks require different information flows within a brain area or between different brain areas. This is due to the fact that neuronal oscillations are background mechanisms essential for dynamic cooperation in the brain [7,8,9,10,11,12].

The most suitable methods for monitoring brain activity to determine functional connectivity are magnetoencephalography (*MEG*) and electroencephalography (*EEG*) due to their good temporal resolutions [13].

Different types of measures can be used to determine functional connectivity, such as phase synchronization, generalized synchronization measures, linear temporal correlation, etc. [14,15,16]. In this paper, we focus on undirected phase synchronization measures. The most often used measures are the phase locking value (*PLV*) [17,18] and the weighted phase lag index (*wPLI*) [19]. The main difference between these two measures is the ability to avoid the effect of volume conduction.

The *PLV* index is based on phase differences of signals from two *EEG* channels. For a set of *N* time points, it calculates an average of *N* unit vectors that represent the phase difference between the signals of both channels. The *PLV* value of zero represents no connection between the observed signals’ regions and the maximum *PLV* value of one represents a perfect connection. Although very widely used, a drawback of the *PLV* measure is the tendency to be biased towards higher values due to volume conduction [17].

The phase lag index (*PLI*) was designed as a solution to avoid the misinterpretation of volume conduction as a connectivity component [17]. Volume conduction reflects in the appearance of signal components with phase differences closer to 0 or ±π. *PLI* avoids them by only considering the number of samples with positive and negative phase differences. Only if the number of samples in one group, i.e., positive or negative, is predominant then *PLI* gets value close to one. This cancels out the components with phase angle distributions centered at 0 and ±π.

The extended version of the *PLI* is the weighted phase lag index (*wPLI* [19]). The *wPLI* measure adds weighting of samples by the imaginary component of the cross-spectral density. Because the real component of cross spectral density is not considered, samples where the phase differences are close to *0* or ±π have no contribution to the connectivity estimation and signal components that may arise due to volume conduction have no influence.

There are several other undirected connectivity measures, such as coherence, imaginary part of coherence, mutual information, etc., but for the purposes of this article, we limit our analysis to only those two most common ones, i.e., *PLV* and *wPLI*. They complement one another, providing connectivity estimation with and without consideration of the volume conduction effect. As an alternative, we propose a complex Pearson correlation coefficient (*CPCC*), which in a single unique measure provides information of both connectivity components.

The rest of the article is structured in the following way. In Section 2, we propose the complex Pearson correlation coefficient as a novel measure of undirected channel connectivity, review the *PLV* and *wPLI* measures, and analytically show their relationships to *CPCC*. In section three, the relationship is demonstrated with practical experiments, using synthetic and real *EEG* signals. We end the paper with a discussion and conclusion.

## 2. Methods

In this section, we define the proposed complex Pearson correlation measure (*CPCC*) and show its analytical relationship with *PLV* and *wPLI* connectivity measures.

### 2.1. Complex Pearson Correlation Coefficient as a Measure of Undirected Connectivity

Various types of complex correlation calculations are used in the literature [20], and in different research fields, such as geophysics [21], radar systems [22], optics [23], etc. In this section, we propose the use of complex Pearson correlation coefficient for *EEG* connectivity analysis.

Pearson’s linear correlation coefficient (*r*) is the most commonly used linear correlation coefficient. It is a statistical measure of the degree to which variables change their values in relation to each other, or in other words, expresses the level to which two variables are linearly related. It is defined as follows:

(1) $$r\left(x_{1},x_{2}\right)=\frac{\sum_{n=1}^{N}\left(x_{1,n}-\bar{x_{1}}\right)\left(x_{2,n}^{*}-\bar{x_{2}^{*}}\right)}{\sqrt{\sum_{n=1}^{N}{\left(x_{1,n}-\bar{x_{1}}\right)}^{2}\sum_{n=1}^{N}{\left(x_{2,n}^{*}-\bar{x_{2}^{*}}\right)}^{2}}}.$$

Here, *N* is the number of samples, $x_{1}$ and $x_{2}$, are the series being analyzed, $\bar{\left\{.\right\}}$ represents mean values of observed series, and ${\left\{.\right\}}^{*}$ is the complex conjugate operator (if the values in series are complex). The resulting *r* ranges from −1 (indicating perfect negative correlation) to +1 (indicating perfect positive correlation). A zero value is an indicator of no linear signal relationship. Assuming that *EEG* signals for the analysis should be pre-filtered, which removes *DC* signal components, the equation can be simplified:

(2) $$r\left(x_{1},x_{2}\right)=\frac{\sum_{n=1}^{N}x_{1,n}\cdot x_{2,n}^{*}}{\sqrt{\sum_{n=1}^{N}{|x_{1,n}|}^{2}}\cdot \sqrt{\sum_{n=1}^{N}{|x_{2,n}|}^{2}}}.$$

The numerator in the Equation (2) can be understood as a time-averaged temporal estimation of sample relationship, while the denominator is a weighting factor to obtain the desired range from −1 to 1. Let us first focus to the numerator. For two oscillatory signals represented as series of real values, the temporal relationship estimation is also an oscillatory signal. Consequently, the temporal contribution of a single time step does not have any direct meaning and at least one period of signal samples need to be averaged to become informative. To improve temporal meaningfulness of the estimation, analytic signal representations can be used instead of real valued ones. Analytic signal sample is a complex number that adds an imaginary part indicating the oscillatory nature of the signal to its existent real valued part. Thus, in addition to a real signal value, the analytic signal sample includes the information of signal instantaneous amplitude and instantaneous phase, which can be represented as a vector in a complex plane. Because basic sinusoidal oscillatory signal keeps the instantaneous amplitude constant over time while its instantaneous phase increases linearly, these vectors are also called phasors. For two phase-locked signals the phase difference is constant and the numerator of Equation (2) gets constant over time, too. Its real value represents the dot product of the phasors, while its imaginary part equals the size of the cross product. Altogether the product of two phasors of two analytic signals is analogous to the cross spectral density for the stationary or quasi-stationary signals. The denominator of Equation (2), needed for scaling, relates to the power of both signals. The final result when using analytic signals is the complex Pearson correlation coefficient (*CPCC*):

(3) $$CPCC=r(x_{a,1},x_{a,2}),$$

where $x_{a}$ denotes analytic signals.

The analytic signal representation is defined only for narrow frequency band signals. In such cases analytic signals can be computed from real ones by adding imaginary part equal to the Hilbert transform (*HT*) of the original signal:

(4) $$x_{a}\left(t\right)=x\left(t\right)+iHT\left(x\left(t\right)\right),$$

where *HT (x(t))* represents the Hilbert transform of *x* (real signal) and $x_{a}\left(t\right)$ is an analytic signal, as explained in [17]. With *HT* we obtain a phasor influenced by all the frequencies in the observed narrow band. Phasors can also be obtained using the discrete Fourier transform, where one phasor presents each frequency component, but only stationary, without temporal dimension. *HT* provides this additional temporal perspective, which enables analysis of non-stationary signals. Because *EEG* signals are non-stationary, in this paper we limit to the analysis of their narrow band pre-filtered components with the analytic form obtained using *HT*.

Connectivity measures estimate the relationship between two signals and this can be performed using the *CPCC*. The in-phase signals have high real *CPCC* part and zero imaginary part. On the other hand, imaginary component represents the relationship between signals with the phase lag of ±π/2. Thus, the connectivity of two brain regions can be estimated considering both parts of the complex *CPCC* value for the corresponding *EEG* signals, by computing its absolute value (*absCPCC*). In such a case the obtained value should be related to the *PLV* value. When the volume conduction effect needs to be avoided, only the imaginary component shall be used (*imCPCC*). Such estimation is expected to be related to the *wPLI* value.

### 2.2. Phase Locking Value PLV and Its Relation to CPCC

Phase-locking value (*PLV*) is calculated based on the phase differences of the two analytical signals [17,18]:

(5) $$PLV_{x_{1},x_{2}}=\left|\frac{1}{N}\sum_{n=1}^{N}e^{i(\Delta \phi_{x_{1,n},x_{2,n}})}\right|.$$

**Figure 1:**
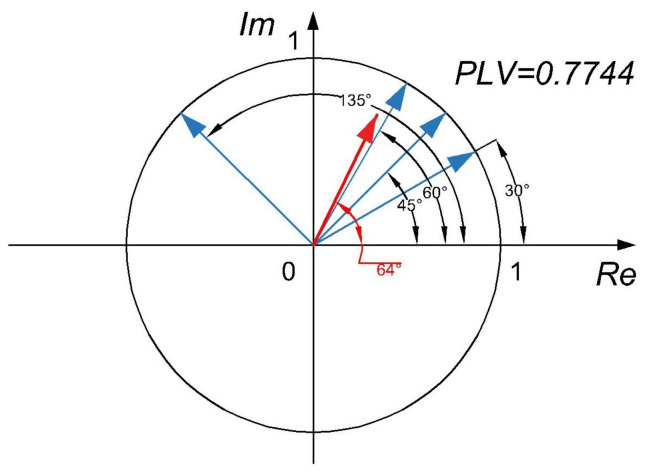
Visualization of averaging used in calculation of the *PLV*. The *PLV* is computed from unit vectors representing instantaneous phase differences.

In Equation (5), Δϕ represents the phase difference and *N* represents the number of samples. The instantaneous phase difference is defined as:

(6) $$\Delta \phi_{x_{1,n},x_{2,n}}=\phi_{x_{1,n}}-\phi_{x_{2,n}},$$

where $\phi_{x_{1,n}}$ and $\phi_{x_{2,n}}$ stand for the phase angles at *n*-th sample. In order to obtain instantaneous phases, analytical signals need to be computed, using (*HT*). Computation of *PLV* can be visualized by creating a set of *N* unit vectors corresponding to *N* time samples, see Figure 1. Phase angles of those vectors are equal to phase differences between the two *EEG* signals for samples from 1 to *N*. All the *N* unit vectors representing phase differences are averaged to obtain *PLV*.

The high value of *PLV* is obtained when the vectors are well clustered, which means that the phase difference between the two *EEG* channels is mostly constant for all the time samples On the other hand, when the phase difference between the two channels is changing with time, the unit vectors are scattered, which results in low *PLV* value.

The lack of this measure is its tendency to falsely over-estimate the connectivity level due to the volume conduction. The reason is that the volume conduction enables a signal from a single source to be measured on both *EEG* electrodes under consideration, which results in a zero-phase difference over a larger time interval, leading to a larger *PLV* value.

In order to prove our assumption that the absolute value of the complex Pearson correlation is related to the *PLV* index, the Equation (2) can be rewritten in the following way:

(7) $$absCPCC_{x_{1},x_{2}}=|r\left(x_{1},x_{2}\right)|=\frac{|\sum_{n=1}^{N}A_{x_{1,n}}\cdot A_{x_{2,n}}\cdot e^{i\Delta \phi_{x_{1,n},x_{2,n}}}|}{\sqrt{\sum_{n=1}^{N}A_{x_{1,n}}^{2}}\cdot \sqrt{\sum_{n=1}^{N}A_{x_{2,n}}^{2}}},$$

where $A_{x}$ represents the instantaneous amplitude of a complex signal.

Comparing Equations (5) and (7), we can see that the *PLV* is related to the *absCPCC*, but scales the contributions of instantaneous phases with instantaneous amplitudes:

(8) $$\frac{|\sum_{n=1}^{N}A_{x_{1,n}}\cdot A_{x_{2,n}}\cdot e^{i\Delta \phi_{x_{1,n},x_{2,n}}}|}{\sqrt{\sum_{n=1}^{N}A_{x_{1,n}}^{2}}\cdot \sqrt{\sum_{n=1}^{N}A_{x_{2,n}}^{2}}}⋄\neq \left|\frac{1}{N}\sum_{n=1}^{N}e^{i(\Delta \phi_{x_{1,n},x_{2,n}})}\right|,$$

*absCPCC* is therefore a weighted version of *PLV*.

### 2.3. Weighted Phase Lag Index wPLI and Its Relation to CPCC

**Figure 2:**
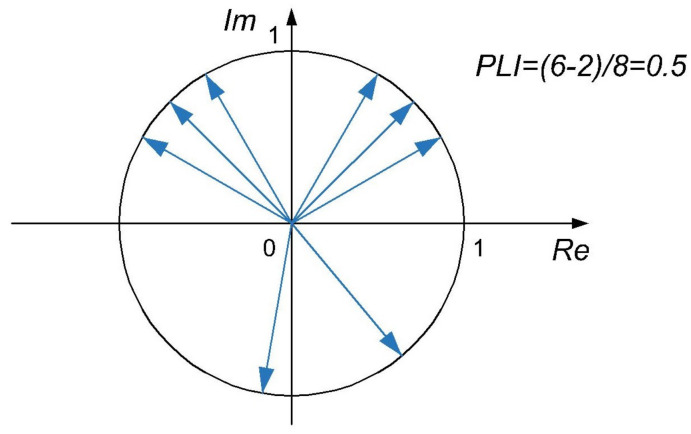
Visualization of averaging used in calculation of the *PLI*. The *PLI* is computed from unit vectors representing instantaneous phase differences.

The *PLI* and *wPLI* measures of connectivity address the volume conduction problem. Let us first present the *PLI* measure, as a transitional step towards a more refined weighted *PLI* measure (*wPLI*). The *PLI* is defined as [17]:

(9) $$PLI_{x_{1},x_{2}}=\left|\frac{1}{N}\sum_{n=1}^{N}sign\left(Im\left(S_{x_{1,n},x_{2,n}}\right)\right)\right|,$$

where *N* is the number of samples. In the original definition [24], $S_{x_{1,n},x_{2,n}}$ is the cross-spectral density of the observed signals defined by Fourier transform. In [17], *PLI* was defined using analytical signals obtained by HT and the cross-spectral density is defined as:

(10) $$S_{x_{1,n},x_{2,n}}=|A_{x_{1,n}}|\cdot |A_{x_{2,n}}|e^{i(\phi_{x_{1,n}}-\phi_{x_{2,n}})},$$

where $A_{x_{1,n}}$ and $A_{x_{2,n}}$ are the instantaneous amplitudes of the observed signals $x_{1}$ and $x_{2}$ at sample *n*. Based on Equation (9) and as shown in, Computation of *PLI* is illustrated in Figure 2. All unit vectors that represent phase differences are first divided into two subsets: those with positive, and those with negative imaginary part. Then, the difference of subsets’ sizes is divided by the number of all vectors *N*, and its absolute value equals the *PLI*.

Therefore, if there is a predominant positive or negative phase difference throughout the observed time interval, then the obtained value of *PLI* will be close or equal to 1. On the contrary, *PLI* which equals 0 is obtained when half of the phase differences are negative and the other half of them are positive.

The weighted phase lag index (*wPLI*) is an improved version of the phase lag index connectivity measure. The unit vectors of phase differences from *PLI* are now scaled with instantaneous amplitudes of both signals [19]. In other words, *wPLI* is obtained by weighting *PLI* with the imaginary part of the cross spectral density:

(11) $$wPLI_{x_{1},x_{2}}=\left|\frac{\frac{1}{N}\sum_{n=1}^{N}|Im\left(S_{x_{1,n},x_{2,n}}\right)|sign\left(Im\left(S_{x_{1,n},x_{2,n}}\right)\right)}{\frac{1}{N}\sum_{n=1}^{N}|Im\left(S_{x_{1,n},x_{2,n}}\right)|}\right|.$$

By expressing the cross spectral density from Equation (10) using the complex conjugate operator, the *wPLI* can be rewritten as follows:

(12) $$S_{x_{1,n},x_{2,n}}=x_{1,n}\cdot x_{2,n}^{*}.$$

(13) $$wPLI_{x_{1},x_{2}}=\left|\frac{\frac{1}{N}\sum_{n=1}^{N}|Im\left(x_{1,n}\cdot x_{2,n}^{*}\right)|sign\left(Im\left(x_{1,n}\cdot x_{2,n}^{*}\right)\right)}{\frac{1}{N}\sum_{n=1}^{N}|Im\left(x_{1,n}\cdot x_{2,n}^{*}\right)|}\right|,$$

which can be further simplified as:

(14) $$wPLI_{x_{1},x_{2}}=\frac{|\sum_{n=1}^{N}Im\left(x_{1,n}\cdot x_{2,n}^{*}\right)|}{\sum_{n=1}^{N}|Im\left(x_{1,n}\cdot x_{2,n}^{*}\right)|}.$$

Now we can show its relationship to the *CPCC* or more specifically to its imaginary part, denoted *imCPCC*:

(15) $$\begin{array}{cc} imCPCC_{x_{1},x_{2}} & =|Im[r\left(x_{1},x_{2}\right)]|\\ \; & =\frac{|Im[\sum_{n=1}^{N}x_{1,n}\cdot x_{2,n}^{*}]|}{\sqrt{\sum_{n=1}^{N}{|x_{1,n}|}^{2}}\cdot \sqrt{\sum_{n=1}^{N}{|x_{2,n}|}^{2}}}\\ \; & =\frac{|\sum_{n=1}^{N}Im\left(x_{1,n}\cdot x_{2,n}^{*}\right)|}{\sqrt{\sum_{n=1}^{N}{|x_{1,n}|}^{2}}\cdot \sqrt{\sum_{n=1}^{N}{|x_{2,n}|}^{2}}} \end{array}$$

Comparing Equations (14) and (15) we see that both measures, *wPLI* and *imCPCC*, are based on the imaginary part of the cross spectral density *S* in the numerator, and differ only in scaling in the denominator. The *wPLI* is scaled using the imaginary part of *S* only, while *imCPCC* with the power of both signals.

### 2.4. Connectivity Estimation Based on Phase Difference Histograms

In this section, we explain how connectivity reflects in phase difference histograms, to illustrate the connectivity measures. Although statistical properties of the phase difference distribution can clearly indicate phase locking of the signals [25], in practice, connectivity measures are rarely explained in these terms. We will use it to gain better insight into real connectivity between signals, particularly for the cases where values of connectivity measures are the highest or most different between each other.

**Figure 3:**
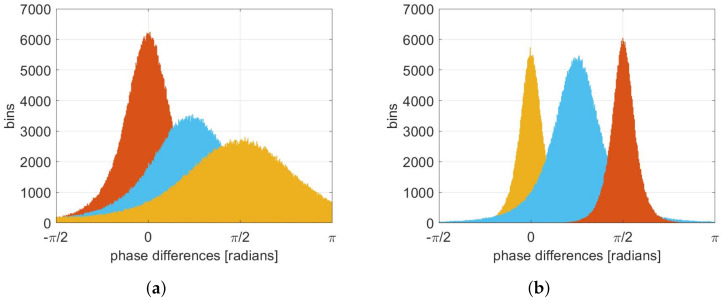
The relationship between connectivity and phase differences distributions. When volume conduction is not considered (**a**) higher connectivity reflects in lower variance, while the mean value is irrelevant. In the presence of volume conduction (**b**), it reflects in higher values for phase differences close to 0 or π, which, therefore, do not (necessarily) indicate connectivity. Connectivity level is expressed with colors; red is the highest and yellow is the lowest.

Let us first assume no volume conduction is present. When two signals are not connected, they change independently and the phase differences are uniformly distributed. Connectivity between two brain regions reflect in more expressed phase differences of corresponding signals. The higher the connectivity, the more pronounced the extreme gets, and the standard deviation of the distribution gets lower, see Figure 3a. The “red” distribution reflects the highest connectivity and its standard deviation is the lowest. On the other hand, the “orange” distribution has the highest standard deviation and reflects the lowest connectivity. The mean value of the phase distribution equals the average phase difference, and can have an arbitrary value in the [−π,π] range, and it does not depend on the connectivity level.

If volume conduction is present, certain signal components are included in both of the signals under consideration. These signal components have a phase difference of 0 or ±π, but due to noise and signal interference, instantaneous phase differences spread around these values. These values therefore do not (necessarily) imply higher connectivity. In the example in Figure 3b, we can expect that distributions with peaks closer to 0±kπ are more likely to reflect volume conduction and not connectivity. The estimated connectivity is therefore the highest when the value at 0 and ±π is the lowest and the variance the smallest.

## 3. Results

In this section, we compare the proposed *CPCC* measures with *PLV* and *wPLI* using synthetic signals and real-life signals from freely available datasets.

### 3.1. Synthetic Signals from the MRC Brain Network Dynamics Unit (University of Oxford)

In the first experiment, we generated synthetic signals following Mäkinen et al. [26]. The *EEG* data we generated contained 31 channels from 973 trials, which were concatenated into a single large signal. This suited our particular purpose, as we analyzed general brain connectivity independent of specific brain events.

**Figure 4:**
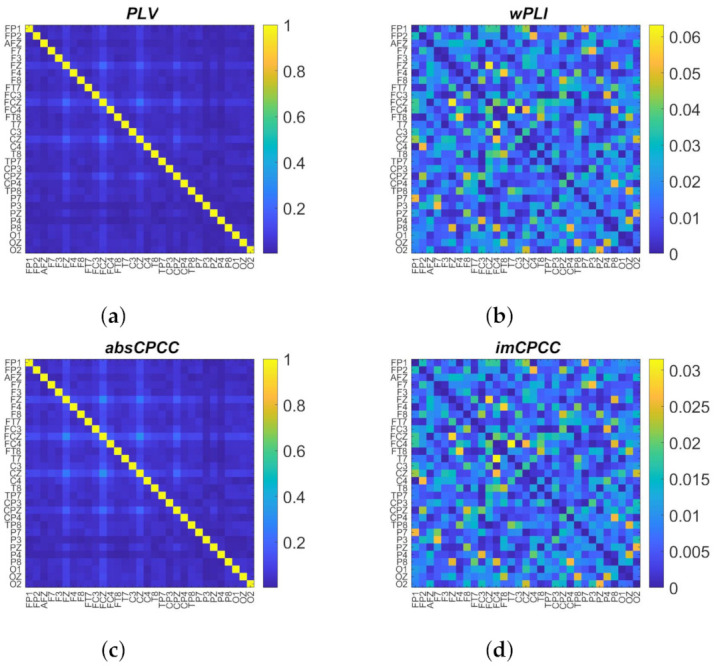
Connectivity matrices obtained with *PLV* (**a**), *wPLI* (**b**), *absCPCC* (**c**), and *imCPCC* (**d**) for signals generated with [26], for 8–13 Hz frequency band.

We computed connectivity using the proposed and established methods for different frequency bands. The connectivity matrices representing the estimated connectivity for each electrode pair and for all four measures are shown in Figure 4. We can clearly see strong visual similarities between the proposed measures and the most commonly used measures, i.e., between *PLV* and *absCPCC*, as well as *wPLI* and *imCPCC*.

**Figure 5:**
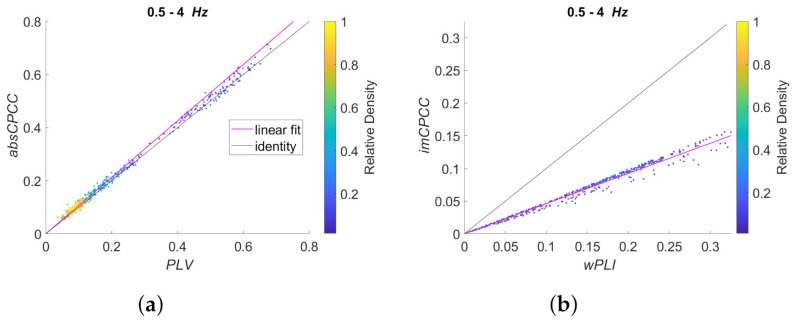

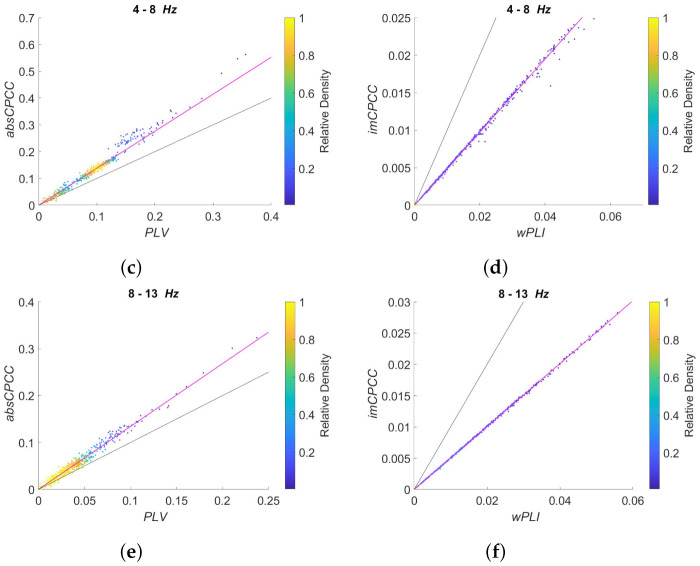
Scatter plots of *absCPCC* to *PLV* relationship (**left**) and *imCPCC* to *wPLI* relationship (**right**). Each dot represents one electrode pair. Dots are colored according to their relative density. The black line represents identity, while the cyan one is the best linear fit. Rows correspond to different frequency bands: (**a**,**b**) 0.5–4 Hz; (**c**,**d**) 4–8 Hz; (**e**,**f**) 8–13 Hz.

To better compare the connectivity measures, see Figure 5, with scatter plots for measure pairs *PLV* to *absCPCC* and *wPLI* to *imCPCC*. Each dot in a scatter plot represents one electrode pair. The color of the dots depends on the relative density of the dots in the graph. There are also two lines shown, where the black one represents identity while the cyan one the best linear fit. High correlation is evident for both connectivity measure pairs, while the scaling differences depend on the frequency band, most evidently for the *PLV* to *absCPCC* pair. There are some electrode pairs that deviate slightly from the general linear relationship while the overall correlation of the measures seem to be high.

To evaluate the relationship between the measures, evident from Figure 5, we computed their correlation. In addition to frequency bands shown in Figure 5, 0.5–4 Hz, 4–8 Hz and 8–13 Hz, we computed it for 13–18, 18–30, and 35–45 Hz frequency bands. For all the frequency bands and both pairs, i.e., *PLV* to *absCPCC*, and *wPLI* to *imCPCC*; the obtained correlation equaled 0.99, proving the close to perfect relationship between the measures.

**Figure 6:**
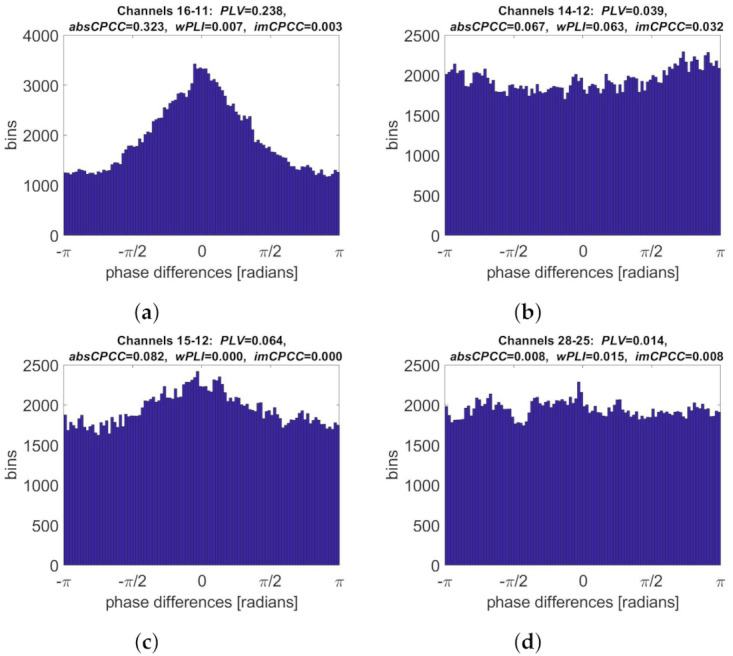
Phase difference distributions for selected electrode pairs (synthetic signals [26]). Shown are the distributions corresponding to the highest: (**a**) *PLV* and *absCPCC* values, (**b**) *wPLI* and *imCPCC* values, (**c**) ratio between *absCPCC* and *imCPCC* values, (**d**) ratio between *imCPCC* and *absCPCC* values.

Finally, we selected electrode pairs with the highest connectivity values and the highest ratio between them. Their phase difference distributions are shown in Figure 6. As expected, the same electrode pair (16–11) had the highest *PLV* and *absCPCC* values. The corresponding phase distribution was centered at the phase angle 0, indicating the possibility of volume conduction. Similarly, one electrode pair (14–12) had the highest in both *wPLI* and *imCPCC* values. The corresponding phase distribution has a less pronounced peak off the center. The ratios between *PLV* and *wPLI*, as well as *absCPCC* and *imCPCC* values, were the highest when the later ones equalled 0 and the histogram was centered (electrode pair 15–12). The highest and *wPLI* to *PLV* ratio and *imCPCC* to *absCPCC* ratio were obtained when *absCPCC* equaled *imCPCC*.

### 3.2. Synthetic Signals Generated with the Kuramoto Model

The second set of synthetic signals to test connectivity estimation methods was generated using the Kuramoto model according to [27]. The reason for using it is that the relationship between electrodes is defined and known in advance. Twenty-four signals (channels/electrodes) were generated. The signals form three groups, from 1 to 8, 9 to 16, and 17 to 24. They are composed of two signal components, where the first component is synchronized between all electrodes in a group and the second component is not synchronized and gives a more realistic variability to the signal set. The signals from 17 to 24 are composed of the first components only and due to the high coupling factor (*K* = 1000), these signals are synchronized very quickly. As a result of the fast synchronization, these signals are in phase and can be observed as an example of high volume conduction.

**Figure 7:**
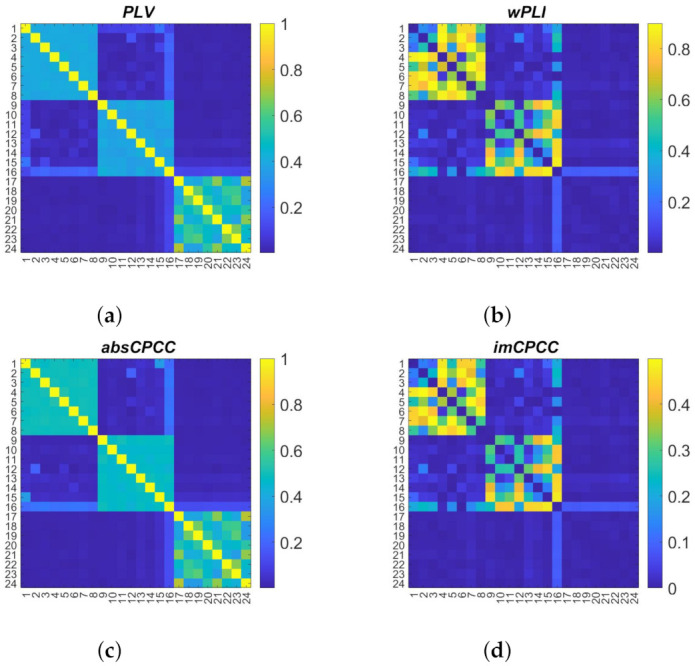
Connectivity matrices obtained with *PLV*, *wPLI*, *absCPCC*, and *imCPCC* for signals generated with the Kuramoto model [27].

Figure 7 shows the connectivity matrices of the observed connectivity measures. It is visible that the signals are connected within groups and much less between the groups. The random nature of the generation process could lead to synchronous signals even between different groups. Looking at the third group of signals, we also see a tendency for the *PLV* and *absCPCC* to include volume conduction as an acceptable contribution to the connectivity, while *wPLI* and *imCPCC* avoid this component.

**Figure 8:**
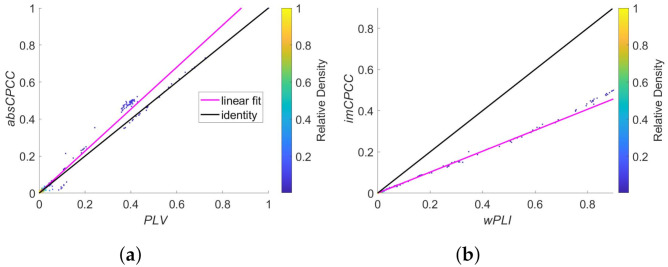
Relationships between *absCPCC* and *PLV* (**a**), and *wPLI* and *imCPCC* (**b**), shown as a scatter plot of values for all signal pairs where signals were generated with the Kuramoto model [27]. The black line represent the identity while the cyan line shows the best linear fit. The colors of the dots represent the relative density of the connectivity values.

In Figure 8 is a scatter plot showing the relationship between *absCPCC* and *PLV* values (a) and between the *imCPCC* and *wPLI* values (b). A strong linear relationship is evident for both connectivity estimation method pairs, while the scaling is different.

The correlation between *PLV* and *absCPCC* values as well as the correlation between *wPLI* and *imCPCC* equals 0.99, indicating strong similarities between these measures.

**Figure 9:**
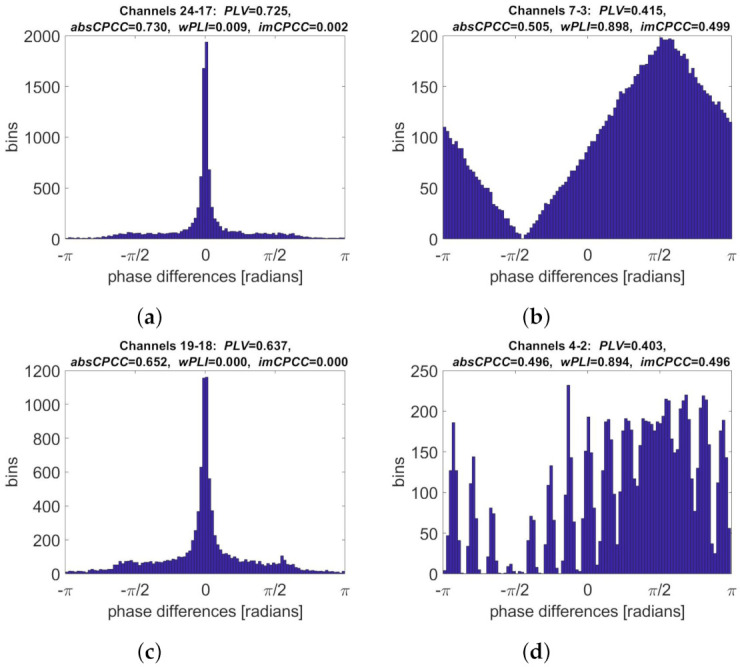
Phase difference distributions for selected synthetic signal pairs generated using the Kuramoto model [27]. Shown are the distributions corresponding to the highest: (**a**) *PLV* and *absCPCC* values, (**b**) *wPLI* and *imCPCC* values, (**c**) ratio between *absCPCC* and *imCPCC* values, (**d**) ratio between *imCPCC* and *absCPCC* values.

The phase difference distributions for signal pairs with the highest connectivity values and the highest ratio between them are shown in Figure 9. The phase difference distribution corresponding to the highest *PLV* and *absCPCC* values was narrow and centered around 0. It was obtained for two signals from the last group (24–17), which modeled volume conduction. The highest *wPLI* and *imCPCC* values were obtained for signals from the first group (7–3), with wide distribution centered at π/2 radians. The ratio between the *absCPCC* and *imCPCC* values was the highest when the latter one equaled 0 and the histogram was centered, indicating possible volume conduction (signal pair 19–18). The highest *imCPCC* to *absCPCC* ratio was, again, obtained when values for both measures were equal, with a clear peak of the distribution at π/2 radians.

### 3.3. Real-Life Signals

For testing on real-life data, we used the SPIS Resting State Dataset [28], a multimodal dataset with *EEG* and forehead *EOG* signals. In our analysis, we used only *EEG* signals from the “eyes closed” (*EC*) and “eyes open” (*EO*) states with a duration of 2.5 min, using 256 Hz sampling rate.

Offline preprocessing of the EEG signal was performed in the following sequence of steps:

1. The raw brain activity data were imported into *MATLAB* using the *EEGLAB* toolbox;
2. Electrode positions (also called channel locations) were defined in the software;
3. The data were referenced to average;
4. The data were filtered with a band pass filter limited to 0.5 and 45 Hz;
5. Automatic spectral-based channel suppression (*z* = 5) was performed using the *EEGLAB “pop rejchan”* function;
6. Artifacts were removed using the *ICLabel* plugin for *EEGLAB* (thresholds for removing components were less than or equal to 0.05 for brain activity and greater than or equal to 0.9 for artifacts);
7. The data were re-referenced to average;
8. Sub-bands of the *EEG* signal were extracted (delta 0.5–4 Hz, theta 4–8 Hz, alpha 8–13 Hz, low beta 13–18 Hz, high beta 18–30 Hz, gamma 35–45 Hz).

**Figure 10:**
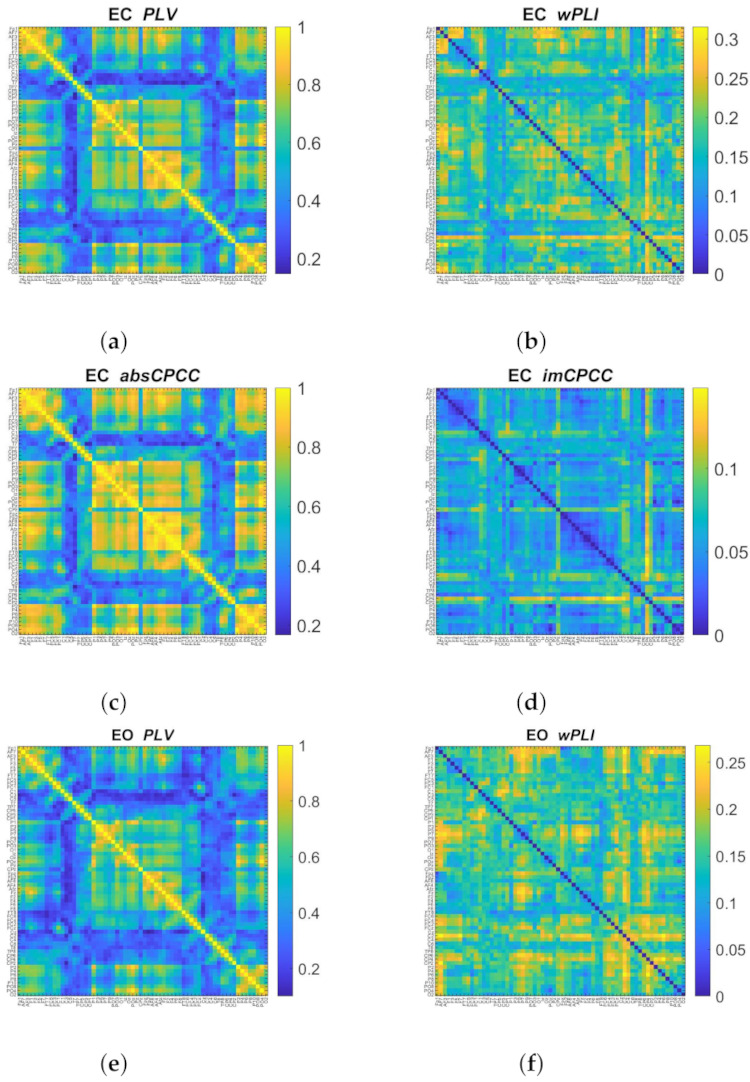

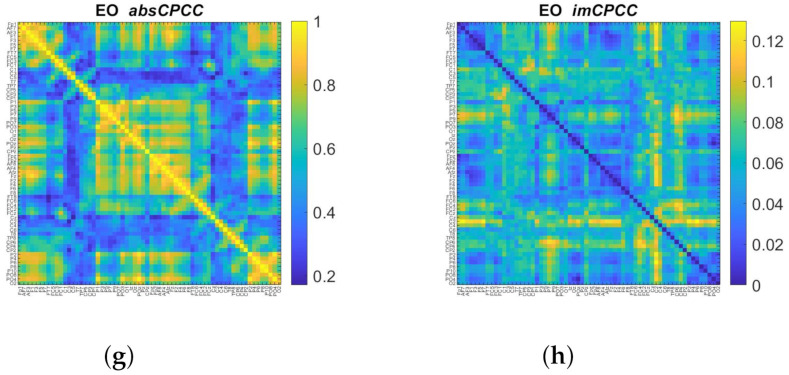
Connectivity matrices for the the alpha band (8–13 Hz) of the real-life signals [28] for eyes closed (EC) and eyes open (EO) states, computed with *PLV*, *wPLI*, *absCPCC* (**g**), and *imCPCC* (**h**).

Figure 10 shows the connectivity matrices for *PLV*, *absCPCC*, *wPLI*, and *imCPCC* for both conditions (*EC* and *EO*) in the alpha band (8–13 Hz). The similarity between *PLV* and *absCPCC*, as well as between *wPLI* and *imCPCC*, is visible, although with some evident differences, mainly between the latter two. Although the patterns are similar, the color scaling (based on the highest value) is different.

**Figure 11:**
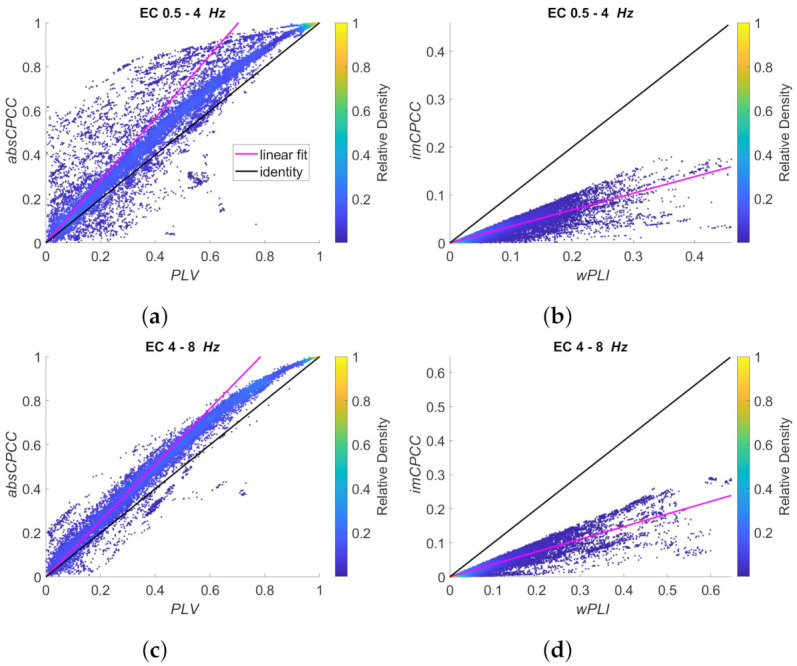

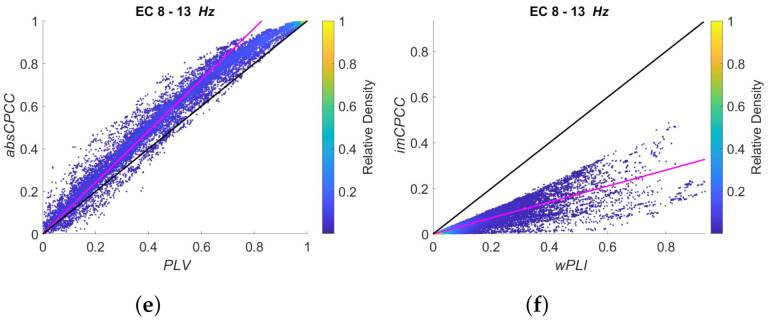
Scatter plots of the *absCPCC* to *PLV* relationship (**left**) and the *imCPCC* to *wPLI* relationship (**right**), for all electrode pairs and for *10* test subjects (*EC* state). The black line represents the identity, while the cyan line shows the best linear fit. The colors of the dots represent the relative density of the connectivity values. Each row is shown for a different frequency band: (**a**,**b**) 0.5–4 Hz; (**c**,**d**) 4–8 Hz; (**e**,**f**) 8–13 Hz.

**Figure 12:**
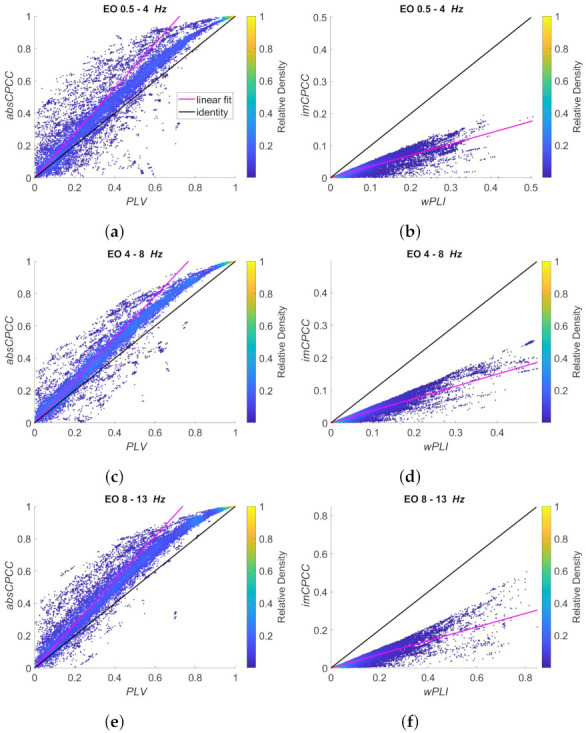
Scatter plots of the *absCPCC* to *PLV* relationship (**left**) and the *imCPCC* to *wPLI* relationship (**right**), for all electrode pairs and for 10 test subjects (*EO* state). The black line represents the identity, while the cyan line shows the best linear fit. The colors of the dots represent the relative density of the connectivity values. Each row is shown for a different frequency band: (**a**,**b**) 0.5–4 Hz; (**c**,**d**) 4–8 Hz; (**e**,**f**) 8–13 Hz.

Figure 11 and Figure 12 show the relationship between *absCPCC* and *PLV* values (left) and between the *imCPCC* and *wPLI* values (right) for all the frequency ranges. Figure 11 shows the signals recorded with eyes closed (EC state), while Figure 12 is for eyes open (EO state). We can see that *absCPCC* and *PLV* as well as *imCPCC* and *wPLI* are positively correlated in all frequency bands. However, we have to be aware that real-life signals include multiple signal components with different amplitudes, while the scaling is common for the whole sequence. This makes the results more scattered in *PLV-absCPCC* and *wPLI-imCPCC* distributions. The relationship between *absCPCC* and *PLV* is evident, but with visible deviation from being perfectly linear due to reduced scaling difference for high connectivity values. The relationship between *imCPCC* and *wPLI* also deviates from linear, with evident range of scaling differences. The relationships do not seem to be dependent on the EC/EO state.

To enumerate the linearity of relationships between the connectivity measures, we show the correlation values between them in Table 1.

The correlation between *absCPCC* and *PLV* connectivity measures is high for all frequency bands and both states (*EC* and *EO*), with an average of 0.97. Only slightly lower values are obtained for the correlation between *imCPCC* and *wPLI*, with an average of 0.92. The corresponding p-values for the alternative hypothesis that measures are not correlated are all smaller than 0.0001 and, thus, well below the significance level of 0.05, which means that the hypothesis of the correlation between the absCPCC and PLV and between imCPCC and wPLI is proven for all frequency bands and both states.

**Figure 13:**
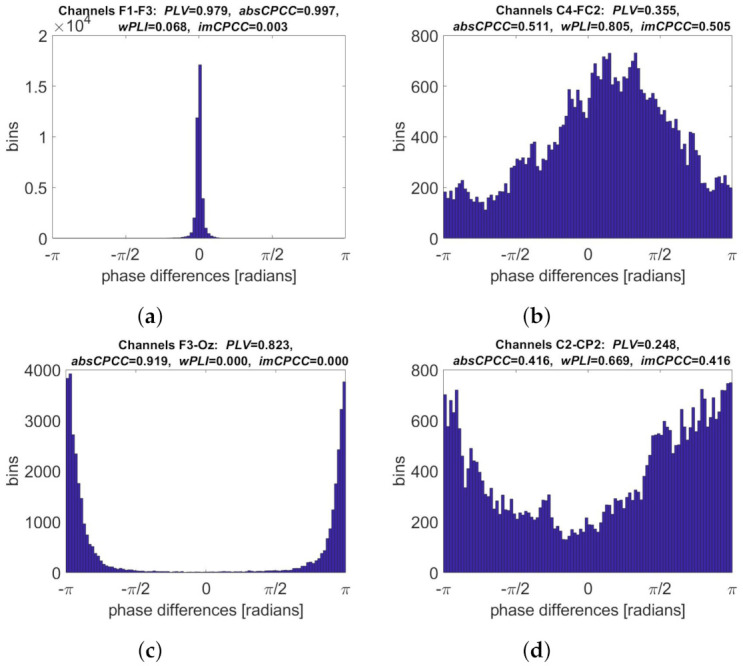
Phase difference distributions for selected electrode pairs (real-life signals [28]). For each distribution, all four measures are calculated. The figure shows the electrode pair with the highest: (**a**) *PLV* and *absCPCC* values, (**b**) *wPLI*, and *imCPCC* values, (**c**) ratio between *absCPCC* and *imCPCC* values, (**d**) ratio between *imCPCC* and *absCPCC* values.

Phase difference distributions for real-life signals for electrode pairs with the highest connectivity values and the highest ratios between them are shown in Figure 13. The phase difference distributions corresponding to the highest *PLV* and *absCPCC* values are narrow and centered around 0. The highest *wPLI* and *imCPCC* values are obtained when the distribution that is wide, slightly asymmetric, and centered at non-zero phase difference. The ratio between *absCPCC* and *imCPCC* values is the highest when the later one equal 0 and the histogram is centered at ±π radians. The highest *imCPCC* to *absCPCC* ratio is, again, obtained when their values are equal and the histogram is not symmetric around 0.

## 4. Discussion and Conclusions

In this paper, we shed new light on the Pearson correlation for the *EEG* connectivity analysis. We introduced the complex correlation (CPCC) as a measure of brain connectivity. We compared it to the (currently) most widely used brain connectivity measures, i.e., *PLV* [17,18] and *wPLI* [19]. The correlation coefficient (CC) has been used before, but only between the real signals, not the analytic ones, and it was shown that it does not represent the optimal metric to estimate functional interactions [29]. It equals the real component of CPCC, while we showed the importance of the absolute value and the imaginary component of CPCC.

We showed that the imaginary part of the complex Pearson correlation (*imCPCC*) is closely related to the *wPLI* measure and that the absolute value of the complex Pearson correlation *absCPCC* is closely related to *PLV*. The relationships are proven analytically and numerically, on two types of synthetic signals [26,27] and on real-life *EEG* signals [28]. Analytically, the differences are only in the denominators that are normalizing the measures to the [0,1] interval. Numerically, high correlations between the results obtained with related measures are shown. For synthetic signals, the correlation level is for all frequency bands equal to 0.99. The scaling differences are evident for *absCPCC* to *PLV* relationship, and differ for different frequency bands. The connectivity results for real-life signals show more differences and the measures are less correlated, but still with an average correlation of 0.97 for *absCPCC* to *PLV* relationship and 0.92 for the *imCPCC* to *wPLI* relationship. Real-life signals consist of more components, which originate in different sources, are related through different neural paths, and include different (although similar) frequencies. Even when limiting frequency bandwidths, they are more information-rich than simulated signals. Connectivity could be understood as a portion of signal components that affects two distinct electrode signals, but can vary with time. All of this reflects in more complex phase difference histograms and more complex scatter-plotted relationships between the measures.

Based on the results shown in this paper, and the fact that connectivity measures are currently typically analyzed relatively, we conclude that *PLV* can be replaced with *absCPCC* and *wPLI* with *imCPCC*. Moreover, the *absCPCC* and *imCPCC* measures are defined as two components of the the same *CPCC* measure and are, therefore, related, while *PLV* and *wPLI* are not. This enables comparison of the connectivity components that may be affected by volume conduction and those which are certainly not. The imaginary component of CPCC can only be lower or equal to the absolute CPCC value due to the excluded real CPCC component, which depends on signal components that may result from volume conduction. It can be expected that the true connectivity may also yield in-phase signals of two electrodes, which makes them indistinguishable from volume conduction. Similar to wPLI, imCPCC scales the components, such that ones more likely arise from volume conduction have lower influence. Thus, the estimated connectivity deviates from the true one, but CPCC provides the upper and lower boundary, with absCPCC and imCPCC respectively. Neuroscientists sometimes have a practice of calculating both the *PLV* and *wPLI* (or *PLI*) and then interpreting the results [29,30,31,32] because the *PLV* method, unlike the *wPLI*, does not take into account the influence of volume conduction. With the proposed *CPCC* measures, they can get additional information, related to the ratio of both connectivity components. As such, the CPCC measure could be used for various neurology related studies. Such studies include the EEG-based brain mechanism of sleep stages, which is important for sleep quality assessment and disease diagnosis [33]. By averaging over the trial set, the proposed measures could also be used as a solution to improve the prediction results of the phases of the synchronization and desynchronization tasks [34]. The potential application of CPCC also lies in the assessment of mental stress levels using functional connectivity as a parameter [35,36] and in the diagnosis dyslexia [37]. In addition, the proposed measures could be used as parameters for the evaluation of simulated EEG data based on the theory of functional connectivity of the brain [38].

The next valuable property of the proposed *CPCC* measure is that it can be computed as a summation of temporal sample contributions. This enables the measure to reveal temporal changes of connectivity and opens a new direction for further research. This can be especially useful for analyzing human brain networks in auditory and visual tasks [39,40] and is also promising for assessing motor skills [41]. This also allows us to observe changes in the organization of brain network connectivity over time using well-known measures from complex network graph theory [42].

The *CPCC* measure has an advantage in the computational complexity. In our experiments the computation of *absCPCC* and *imCPCC* was 65% to 179% faster than the computations of *PLV* and *wPLI*.

Finally, the computation of the correlation of two analytic signals is easy to implement and already available in most of the statistical and signal analysis tools.

Following the above discussion—we can state that the newly proposed *CPCC* connectivity measure, with *absCPCC* and *imCPCC* as its components, could replace *PLV* and *wPLI* measures, accelerate the computation of brain connectivity, and provide further information about brain processes. The data, code, and instructions for replicating the study presented in this article are freely available at https://github.com/zsverko/Code_CPCC.git (accessed on 10 January 2022).

## Figures and Tables

**Table 1:** Correlation values between compared connectivity measures (real-life signal). Here, $r_{abs}$ and $r_{im}$ denote r(absCPCC,PLV) and r(imCPCC,wPLI) respectively.

Frequency	State-EC		State-EO	
**(Hz)**	$r_{\mathrm{abs}}$	$r_{\mathrm{im}}$	$r_{\mathrm{abs}}$	$r_{\mathrm{im}}$
0.5–4	0.93	0.86	0.94	0.89
4–8	0.98	0.91	0.97	0.94
8–13	0.98	0.86	0.97	0.91
13–18	0.99	0.94	0.99	0.96
18–30	0.98	0.96	0.99	0.96
35–45	0.96	0.95	0.99	0.95

## Data Availability

The data, code, and instructions are freely available at https://github.com/zsverko/Code_CPCC.git (accessed on 10 January 2022).

## Abbreviations

The following abbreviations are used in this manuscript:

absCPCC	absolute value of complex Pearson correlation coefficient
CPCC	complex Pearson correlation coefficient
DTI	diffusion tensor imaging
EC	eyes closed
EEG	electroencephalography
EO	eyes open
HT	Hilbert transform
imCPCC	imaginary component of complex Pearson correlation coefficient
MEG	magnetoencephalography
MRI	magnetic resonance imaging
PLV	phase locking value
PLI	phase lag index
wPLI	weighted phase lag index
